# Flow and Heat Transfer in Sisko Fluid with Convective Boundary Condition

**DOI:** 10.1371/journal.pone.0107989

**Published:** 2014-10-06

**Authors:** Rabia Malik, Masood Khan, Asif Munir, Waqar Azeem Khan

**Affiliations:** Department of Mathematics, Quaid-i-Azam University, Islamabad, Pakistan; China University of Mining and Technology, China

## Abstract

In this article, we have studied the flow and heat transfer in Sisko fluid with convective boundary condition over a non-isothermal stretching sheet. The flow is influenced by non-linearly stretching sheet in the presence of a uniform transverse magnetic field. The partial differential equations governing the problem have been reduced by similarity transformations into the ordinary differential equations. The transformed coupled ordinary differential equations are then solved analytically by using the homotopy analysis method (HAM) and numerically by the shooting method. Effects of different parameters like power-law index 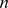, magnetic parameter 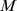, stretching parameter 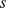, generalized Prandtl number Pr and generalized Biot number 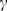 are presented graphically. It is found that temperature profile increases with the increasing value of 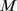 and 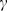 whereas it decreases for 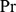. Numerical values of the skin-friction coefficient and local Nusselt number are tabulated at various physical situations. In addition, a comparison between the HAM and exact solutions is also made as a special case and excellent agreement between results enhance a confidence in the HAM results.

## Introduction

Because of the occurrence in a variety of engineering operations the boundary layer flow and heat transfer over a stretching surface has gained much importance. A few applications in the field of chemical engineering and metallurgy include extrusion of polymers, production of paper and so forth. The final product's quality massively depends on heat transfer rate between the fluid and stretching surface during the operation of heating and/or cooling. Consequently, most suitable heating and/or cooling fluid must be chosen as it has immense influence on the heat transfer rate. The physical importance of heat transfer over a moving surface has compelled many researchers to report their findings on this topic [1]–[10].

The convective heat transfer is of excessive significance in procedures in which high temperatures are involved. For instance, gas turbines, nuclear plants, storage of thermal energy etc. Referring to numerous industrial and engineering processes the convective boundary conditions are more practical including material drying, transpiration cooling process etc. Due to the practical importance of convective boundary conditions several researchers have studied and reported results on this topic for viscous fluid. Bataller [11] investigated the Blasius and Sakiadis flows in a viscous fluid with convective boundary conditions. The heat transfer of a viscous fluid over a stretching/shrinking sheet with convective boundary conditions has been studied by Yao *et al.* [12]. Hammad *et al.* [13] discussed the radiation effects and effects of the thermal convective boundary condition, variable viscosity and thermal conductivity on coupled heat and mass transfer with mixed convection. Vajravelu *et al.* [14] presented solution to the unsteady convective boundary layer flow of a viscous fluid over a vertical stretching surface with thermal radiation.

On the other hand, the study of non-Newtonian fluids including Generalized Newtonian Liquid (abbreviated as GNL) with heat transfer has gained extensive importance due to a number of industrial applications such as molten plastic, polymer solutions, pulp and foods etc. At the same time, heat transfer in non-Newtonian fluids with convective boundary conditions has been dealt by a few researchers. The three-dimensional flow of a Jeffrey fluid over a stretching surface with convective boundary conditions has been examined by Hayat *et al.* [15]. In another paper, the flow and heat transfer in an upper-convected Maxwell fluid over a moving surface in the presence of a free stream velocity with convective boundary conditions is studied by Hayat *et al.* [16]. The steady flow and heat transfer in an Eyring Powell fluid over a plate moving continuously concerning convective boundary conditions is also examined by Hayat *et al.* [17]. Srinivas *et al.* [18] examined the influence of chemical reaction and Soret effects on hydromagnetic viscous pulsating flow in a porous channel with convective boundary conditions. Makinde [19] analyzed the thermal stability of viscous fluid flowing steadily through a channel filled with the saturated porous medium. The Sisko model [20], [21] a special case of GNL which predicts dilatant and pseudoplastic nature of fluid is not given due attention. It is worth pointing out that a few recent investigations on flow of Sisko fluid with heat transfer have been studied by Khan and Farooq [22] and Khan *et al.* [23], [24].

However we can notice that the Sisko fluid with heat transfer analysis specially with an emphasis of convective boundary conditions is less explored. In the work under consideration we explored the flow and heat transfer in Sisko fluid over a nonlinearly stretching surface with convective boundary condition. It is hoped that present work serves as stimulus for the shear thinning and thickening fluid flows in the areas where high rate of heat transfer or rate of cooling is required such as extrusion processes, glass fiber and storage of thermal energy.

## Mathematical Formulation

### Flow equations

**Figure 1:**
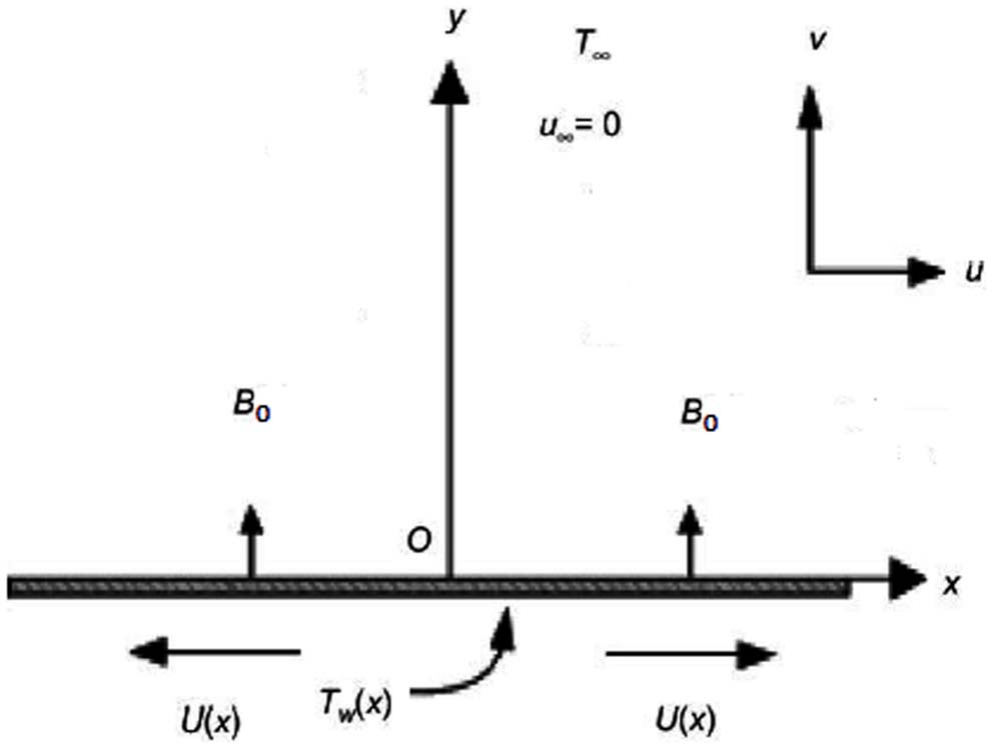
Schematic diagram of the problem.

Let us consider steady, laminar and incompressible flow of Sisko fluid over an isothermal flat sheet (as shown in figure 1). The sheet is stretching with velocity 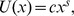 where 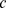 and 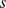 are non-negative real numbers and the velocity for two-dimensional flow is assumed of the form 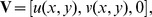 where 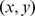 denotes the Cartesian coordinates. A uniform transverse magnetic field 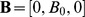 is applied under the assumption of very small magnetic Reynolds number. The governing equations for two-dimensional boundary layer flow are (see ref. [20] for details)
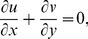
(1)

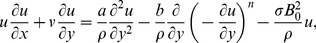
(2)where 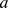, 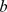 and 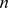 (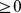) are the material constants, 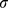 the electrical conductivity of the fluid, 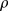 the fluid density, 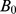 the magnitude of applied magnetic field.

The flow is subject to the following boundary conditions
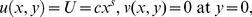
(3)

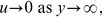
(4)where 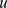 and 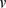 are the velocity components along 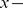 and 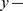 directions, respectively.

Introducing the transformations [20] as
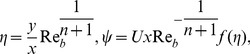
(5)with
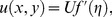
(6)

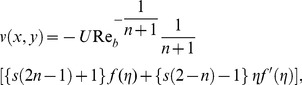
(7)where 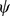 is the Stokes stream function.

After simplification we reach at the following problem [20]

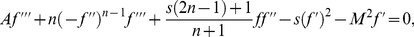
(8)

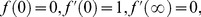
(9)where
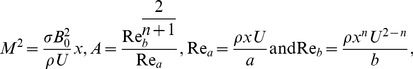
(10)are the non-dimensional quantities.

The significant quantity of interest is the skin-friction 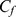 given by [8]

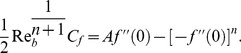
(11)


### Heat transfer analysis

The thermal energy equation after the application of usual thermal boundary layer approximation in the absence of heat source and dissipation with convective boundary condition at the wall is given as
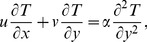
(12)

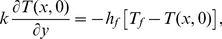
(13)

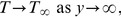
(14)where 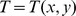 is the temperature field, 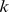 the thermal conductivity, 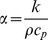 the thermal diffusivity, 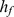 the heat transfer parameter and 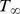 the ambient temperature of the fluid.

We introduce the non-dimensional scaled temperature 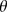 as
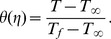
(15)Using Eqs. (5) and (15) Eq. (12) takes the form
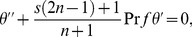
(16)and transformed boundary conditions are
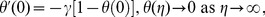
(17)where prime denotes differentiation with respect to 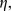 
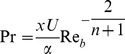
 the generalized Prandtl number and 
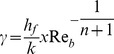
 the generalized Biot number.

The local Nusselt number 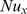 may be found in terms of the dimensionless temperature at the wall surface, 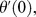 that is
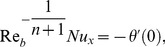
(18)with 
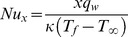
 with 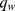 as the surface heat flux.

## Solution Methodology

### The homotopy analytic solution

The homotopy analysis method (HAM) is employed to solve non-linear Eqs. (8) and (16) subject to the boundary conditions (9) and (17) respectively. The analytic solutions are obtained for the velocity and temperature fields. The convergence of these solutions is ensured by taking the most suitable value of the auxiliary parameter 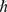 which is calculated using the squared residual error in each case of our calculations, where formula for squared residual error is given by [25]

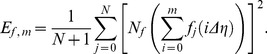
(19)
Table 1 elucidates the convergence of series solution. It shows that the convergence is achieved at 25th approximation in the mentioned case. Further, the same criteria are adopted to achieve the convergence in other cases.

**Table 1:** The convergence of the homotopy solutions when 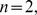 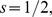 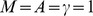 and 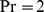 are fixed.

Order of approximation	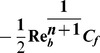	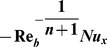
1	1.609750	0.463171
5	1.629799	0.448468
10	1.631518	0.451261
15	1.631523	0.450955
21	1.631523	0.450998
24	1.631523	0.450993
27	1.631523	0.450994
30	1.631523	0.450994

### Exact solutions for particular cases

#### Case (i)

As a special case of the problem for 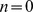 and 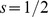 Eqs. (8) and (16) reduce to
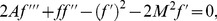
(20)and
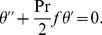
(21)The exact solutions of the above equations satisfying the boundary conditions (9) and (17) are (see ref. [20])
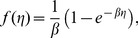
(22)

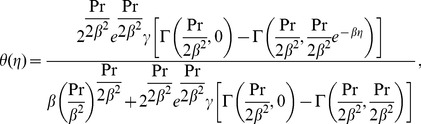
(23)where 
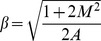
 and 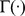 the incomplete Gamma function.

#### Case (ii)

Now for 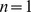 and 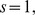 Eqs. (8) and (16) become
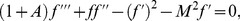
(24)and
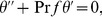
(25)which possess the exact analytical solutions of the form (see ref. [20])
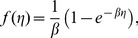
(26)

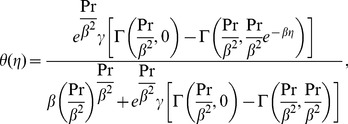
(27)with 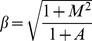.

## Numerical Results and Discussion

In order to get definite perception of the physical problem, velocity profile 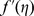 and temperature profile 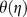 are displayed graphically for different values of the power-law index 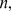
magnetic parameter 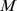, stretching parameter 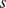, generalized Prandtl number Pr and generalized Biot number 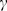 appearing in the problem. The coupled set of Eqs. (8) and (16) with the boundary conditions (9) and (17) are solved analytically by means of the HAM and numerical solutions are obtained using the shooting method. Further, it is possible in some special cases to compare the results obtained by the HAM with exact solutions. Moreover, representative results for the skin-friction coefficient and local Nusselt number illustrating the influence of various physical parameters of the flow are recorded through tables.

**Figure 2:**
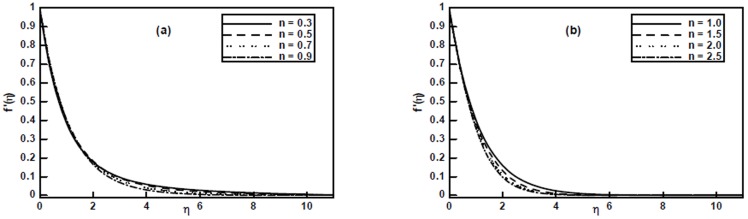
The velocity profiles 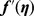 for different values of the power-law index 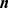 when 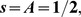 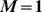 are fixed.

Taking into account the obtained numerical solutions, figures 2(a, b) delineate the influence of the non-integer power-law index 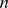 on velocity profile 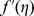. From these figures, it is observed that an increase in the values of 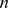 decreases the velocity profile and hence the boundary layer thickness for power index 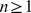 whereas for 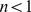 we notice two different behaviors, i.e., close to the sheet the velocity profile increases while it decreases away from the sheet with the increase of the power-law index 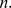


**Figure 3:**
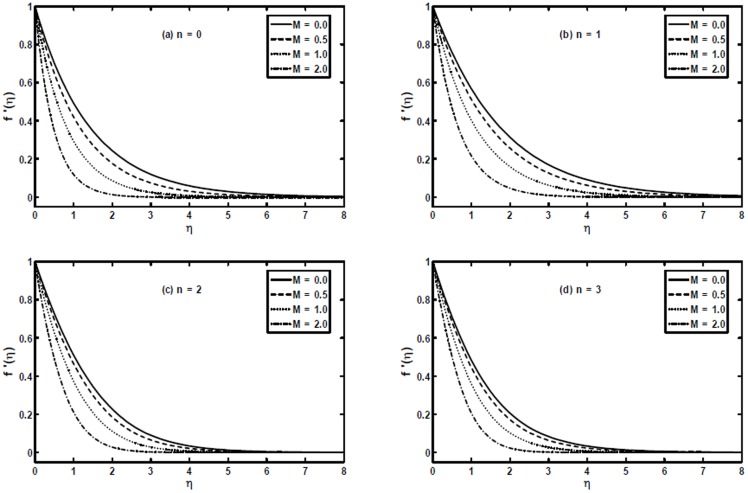
The velocity profiles 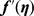 for different values of the magnetic parameter 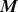 when 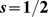 and 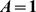 are fixed.

In order to illustrate the influence of the magnetic parameter 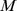 on velocity profile 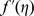 we have plotted figures 3(a-d) for the power-law index 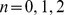 and 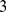. It appears from these figures that an increase in value of the magnetic parameter 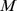 decreases the velocity profile due to resistance force generated by the magnetic field. Also, we can notice that effect of the magnetic parameter 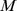 becomes less dominating as we increase value of the power-law index 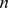 and boundary layer thickness decreases with the increase of 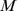 too. Further, these figures portray that the boundary layer thickness becomes thin as we decrease the power-law index 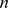. Moreover, these figures provide a comparison that the magnitude of velocity is larger for hydrodynamic case (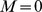) when compared with hydromagnetic case (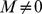).

**Figure 4:**
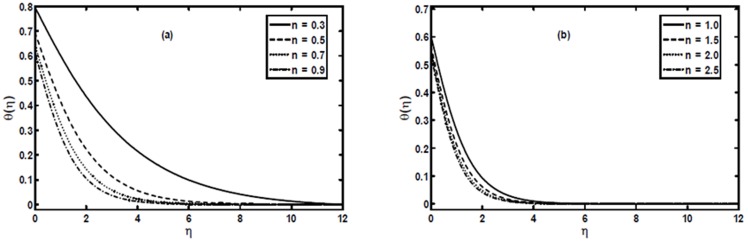
The temperature profiles 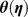 for different values of the power-law index 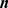 when 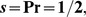 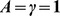 and 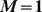 are fixed.

Figures 4(a, b) correspond to the numerical solution obtained for the non-integer power-law index 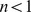 and 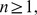 respectively. From these figures, it is obvious that the temperature profile decreases with increase in the power-law index 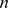. Further, these figures indicate that for a given location 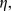 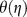 decreases as the power-law index 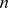 increases, resulting in a decrease of the thermal boundary layer thickness. We can also observe that more significant effects can be seen for values of the power-law index 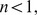 while it has small effects for the power-law index 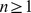.

**Figure 5:**
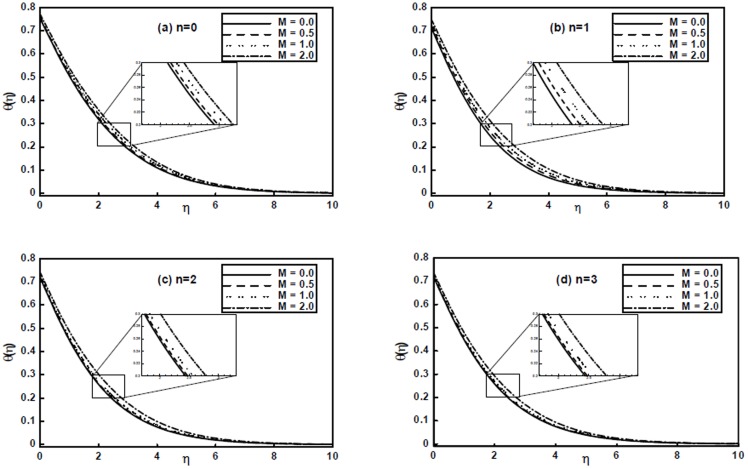
The temperature profiles 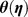 for different values of the magnetic parameter 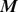 when 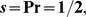 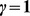 and 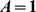 are fixed.

Figures 5(a–d) portray the effects of the magnetic parameter 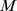 on temperature profile 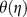. It is clear from these figures that the temperature profile increases with an increase of 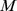. However, we can observe that the temperature profile is not very much sensitive to the magnetic parameter 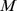.

**Figure 6:**
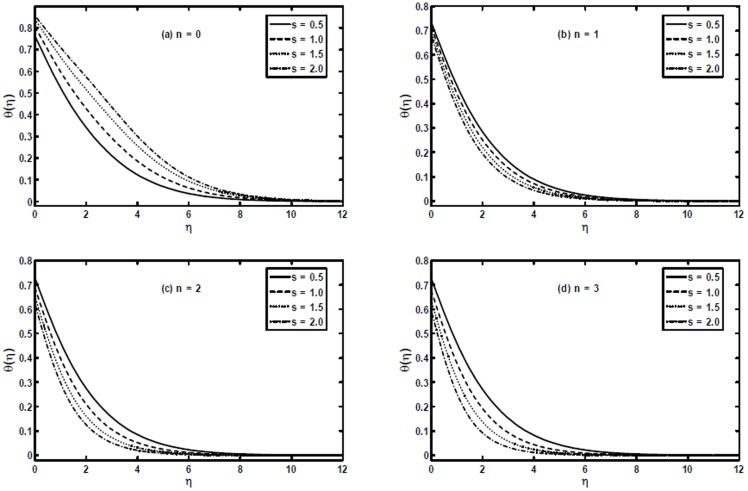
The temperature profiles 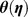 for different values of the stretching parameter 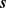 when 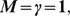 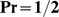 and 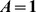 are fixed.

Figures 6(a–d) present the temperature profile 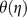 for different values of the stretching parameter 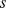. We can notice from these figures that the stretching parameter has quite opposite effect on the temperature profile for 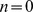 and 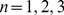. We can see that with an increase in the stretching parameter 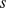 the temperature profile increases for 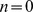, while for 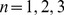 it decreases. Further, with the increase in 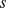 the thermal boundary layer thickness increases for 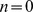 and decreases for 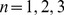.

**Figure 7:**
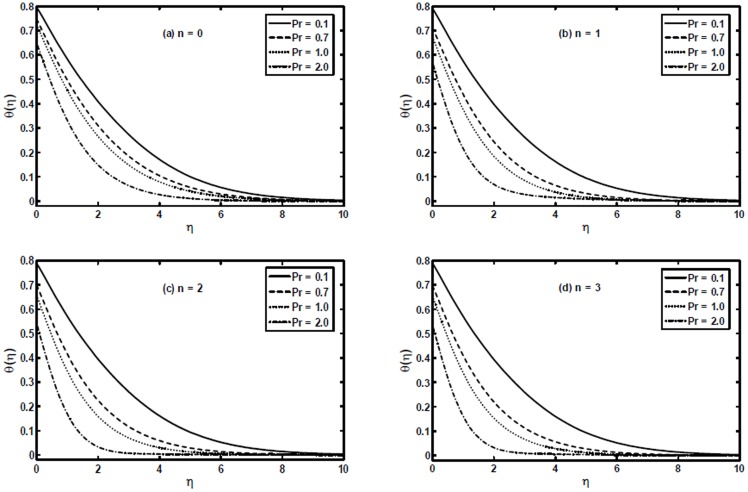
The temperature profiles 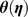 for different values of the generalized Prandtl number 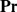 when 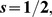 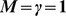 and 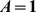 are fixed.

The variation of the generalized Prandtl number 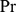 on the temperature profile 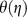 is shown in Figures 7(a–d). It is worth noting that with the increase of 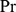 the temperature profile decreases. That is, an increase in generalized Prandtl number 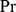 results in a decrease in the thermal conductivity which as a result reduces the thermal boundary layer thickness. Additionally, it can be observed that the power-law index 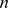 plays a significant role. An increase in the power-law index 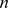 results in thinning of the thermal boundary layer.

**Figure 8:**
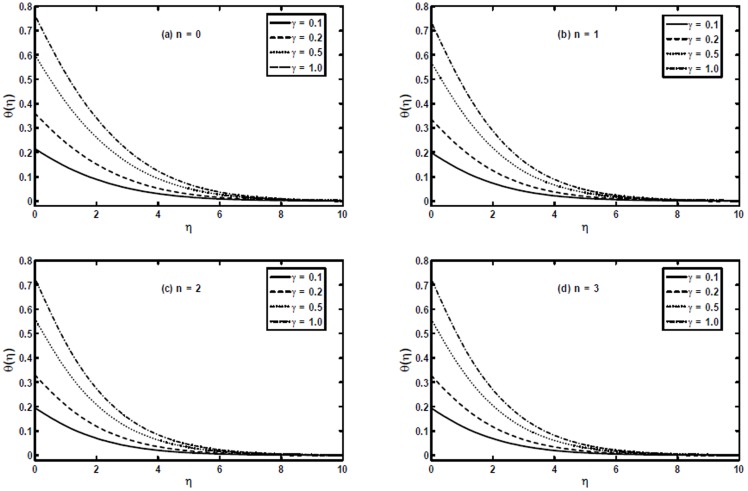
The temperature profiles 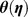 for different values of the generalized Biot number 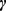 when 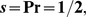 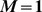 and 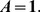

Figures 8(a–d) show the effect of the generalized Biot number 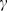 on the temperature profile 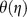. These figures put in evidence that the effect of increasing the generalized Biot number 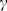 is to enhance both the temperature and thermal boundary layer thickness significantly. It is due to the fact increasing values of 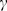 shows the decreasing thermal resistance of the wall and hence convective heat transfer to the fluid increases.

**Figure 9:**
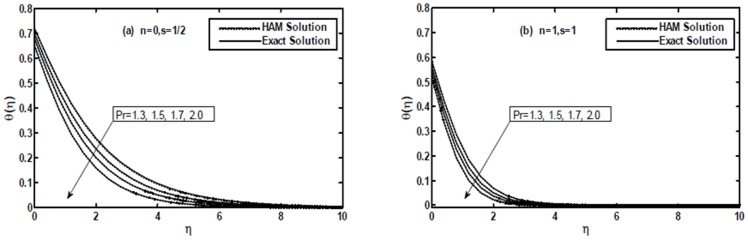
The comparison of the HAM solution with exact solution for the temperature profile 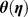 when 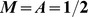 and 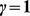 are fixed.

**Figure 10:**
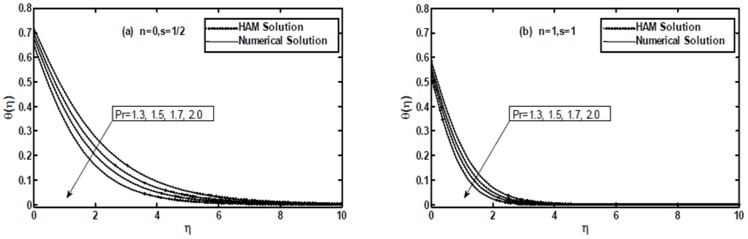
The comparison of the HAM solution with numerical solution for the temperature profile 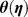 when 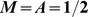 and 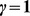 are fixed.

Figures 9(a, b) and 10(a, b) present a comparison between the exact, numerical and HAM solutions. These figures show that excellent agreement between the results exists. This leads confidence in the HAM results reported in this section.

The numerical values of the skin friction coefficient 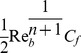 and local Nusselt number 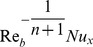 for different values of *A, M, s,* Pr and 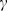 are listed in tables 2 and 3. Table 2 shows that magnitude of the skin friction coefficient increases for larger values of *A, M* and *s*. Table 3 depicts that the local Nusselt number increases for larger values of *A*, Pr, 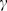 while it has opposite behavior for *M* for different values of the power-law index 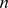. By increasing the stretching parameter 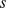 we observe that for 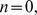 the local Nusselt number decreases while for 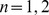 and 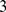 it increases.

**Table 2:** Numerical values of the skin friction coefficient 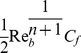 for different values of physical parameters.

*A*	*M*	*s*	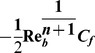			
			*n* = 0	*n* = 1	*n* = 2	*n* = 3
0.0	1.0	0.5	1.000000	1.259683	1.168175	1.121380
1.0			2.224745	1.781461	1.631523	1.558845
2.0			2.732051	2.181835	2.024553	1.954535
3.0			3.121320	2.519366	2.366378	2.304718
1.0	0.0		1.707107	1.089465	0.962186	0.908237
	0.5		1.866025	1.296307	1.153318	1.090619
	1.0		2.224745	1.781461	1.631523	1.558845
	2.0		3.121320	3.028033	2.999168	2.981083
	1.0	0.5	2.224745	1.781461	1.631523	1.558845
		1.0	2.290994	2.00000	1.914495	1.875081
		2.0	2.431934	2.376857	2.412204	2.446648
		3.0	2.573536	2.701216	2.851608	2.965925

**Table 3:** Numerical values of the local Nusselt number 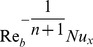 for different values of physical parameters.

*A*	*M*	*Pr*	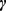	*s*	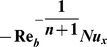			
					*n* = 0	*n* = 1	*n* = 2	*n* = 3
1.0	1.0	0.0	1.0	0.5	0.240458	0.331260	0.340045	0.342428
2.0					0.275129	0.346666	0.357093	0.360945
3.0					0.291544	0.355742	0.367068	0.371748
1.0	0.0				0.291544	0.362207	0.367377	0.368707
	0.5				0.275129	0.353119	0.359256	0.360679
	1.0				0.240458	0.331260	0.340045	0.342428
	1.0	0.7			0.191051	0.277793	0.284027	0.285370
		1.0			0.240458	0.353119	0.340045	0.342428
		2.0			0.349286	0.438483	0.450994	0.455287
		1.0	0.1		0.075993	0.083203	0.083747	0.083891
			0.5		0.193846	0.248832	0.253757	0.255081
			1.0		0.240458	0.331260	0.340045	0.342428
			1.0	0.5	0.240458	0.331260	0.340045	0.342428
				0.7	0.173867	0.347092	0.370199	0.378838
				1.0	0.024606	0.367879	0.406270	0.420928

## Conclusions

In this study, we have investigated the heat transfer with convective boundary condition at the wall for Sisko fluid flow over a non-linearly stretching sheet in the presence of a transverse uniform magnetic field. The governing non-linear equations were formulated and solved analytically by the HAM and numerically by shooting method. Additionally, the exact analytical solutions have been determined for the power-law index 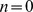 and 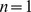. The obtained results imply the following pronouncements.

- For the power-law index 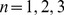 the velocity profile as well as boundary layer thickness was decreased for stretching parameter 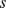 whereas, for 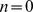 boundary layer thickness was increased.
- Behavior of the material parameter 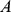 and magnetic parameter 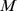 on velocity profile were quite opposite.
- Behavior of stretching parameter 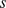 for the temperature profile was similar to that of velocity profile qualitatively.
- The influence of 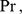 and 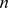 was to decrease the temperature field 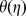 and hence decreased the thermal boundary layer while it increased for 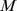 and 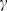.
- For the increasing power-law index 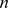 velocity profile as well as temperature profile was decreased and these effects were more noticeable when considering 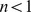 as compared to 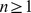

It is expected that the present analysis serves as stimulus for the shear thinning and thickening fluid flows in the areas where high rate of heat transfer or rate of cooling is required such as extrusion processes, glass fiber and storage of thermal energy.
